# Miniaturized microfluidic-based nucleic acid analyzer to identify new biomarkers of biopsy lung cancer samples for subtyping

**DOI:** 10.3389/fchem.2022.946157

**Published:** 2022-08-29

**Authors:** Xue Lin, Zi-Hao Bo, Wenqi Lv, Zhanping Zhou, Qin Huang, Wenli Du, Xiaohui Shan, Rongxin Fu, Xiangyu Jin, Han Yang, Ya Su, Kai Jiang, Yuchen Guo, Hongwu Wang, Feng Xu, Guoliang Huang

**Affiliations:** ^1^ Department of Biomedical Engineering, School of Medicine, Tsinghua University, Beijing, China; ^2^ BNRist and School of Software, Tsinghua University, Beijing, China; ^3^ Beijing National Research Center for Information Science and Technology, Tsinghua University, Beijing, China; ^4^ Dongzhimen Hospital, Beijing University of Chinese Medicine, Beijing, China; ^5^ Emergency General Hospital, Beijing, China; ^6^ National Engineering Research Center for Beijing Biochip Technology, Beijing, China

**Keywords:** lung cancer, microfluidic chip, AI diagnosis, nucleic acid biomarker, loop-mediated isothermal amplification

## Abstract

Identifying new biomarkers is necessary and important to diagnose and treat malignant lung cancer. However, existing protein marker detection methods usually require complex operation steps, leading to a lag time for diagnosis. Herein, we developed a rapid, minimally invasive, and convenient nucleic acid biomarker recognition method, which enabled the combined specific detection of 11 lung cancer typing markers in a microliter reaction system after only one sampling. The primers for the combined specific detection of 11 lung cancer typing markers were designed and screened, and the microfluidic chip for parallel detection of the multiple markers was designed and developed. Furthermore, a miniaturized microfluidic-based analyzer was also constructed. By developing a microfluidic chip and a miniaturized nucleic acid analyzer, we enabled the detection of the mRNA expression levels of multiple biomarkers in rice-sized tissue samples. The miniaturized nucleic acid analyzer could detect ≥10 copies of nucleic acids. The cell volume of the typing reaction on the microfluidic chip was only 0.94 μL, less than 1/25 of that of the conventional 25-μL Eppendorf tube PCR method, which significantly reduced the testing cost and significantly simplified the analysis of multiple biomarkers in parallel. With a simple injection operation and reverse transcription loop-mediated isothermal amplification (RT-LAMP), real-time detection of 11 lung cancer nucleic acid biomarkers was performed within 45 min. Given these compelling features, 86 clinical samples were tested using the miniaturized nucleic acid analyzer and classified according to the cutoff values of the 11 biomarkers. Furthermore, multi-biomarker analysis was conducted by a machine learning model to classify different subtypes of lung cancer, with an average area under the curve (AUC) of 0.934. This method shows great potential for the identification of new nucleic acid biomarkers and the accurate diagnosis of lung cancer.

## 1 Introduction

According to the American Cancer Society, the incidence of lung cancer will be the second-highest among all cancers in 2021, and the mortality rate will be first (Siegel et al., 2021). In China, lung cancer is the most commonly diagnosed cancer type and was the most common cause of cancer-related death in 2018 (Feng et al., 2019). According to its main histotype, prognostic, and therapeutic implications, lung cancer is divided into two main groups: non-small-cell lung cancer (NSCLC) and small-cell lung cancer (SCLC), with the majority being NSCLC (Fujimoto and Wistuba, 2014). Among all the types of NSCLC, lung adenocarcinoma (LUAD), lung squamous cell carcinoma (LUSC), and large cell carcinoma are the three main types. The classification of NSCLC and SCLC is a significant reference for the choice of treatment methods (Harmsma et al., 2013). Furthermore, specific subtyping, especially between LUAD and LUSC, is crucial to the selection of anti-cancer drugs and individualized treatment (Sandler et al., 2006).

As the gold standard for a definite diagnosis, the pathological sectioning method detects lung cancer protein biomarkers. Tumor biomarkers are produced and secreted by tumor cells during the oncogenesis and development of malignant tumors (Ren and Zhao, 2014). In an effort to detect biomarkers, various methods and strategies based on biochemistry (Kondo, 2019), immunology (Arya and Estrela, 2018), and molecular biology (Parida et al., 2005; Maeda et al., 2009) are continuously being verified, developed, and used. Nevertheless, because of their targets being proteins, these methods usually require a large sample volume, complicated manual processing steps, and a lengthy time (1 week) to obtain final results. In addition, it remains challenging to precisely classify the complex subtypes.

The “central dogma” indicates that mRNA is the precursor of protein, and mRNA expression is correlated with protein levels (Li et al., 2014). Considering the relative simplicity of nucleic acid detection, we chose mRNA as our detection target. Recently, some research has focused on the detection of biomarkers in body fluids because of their accessibility and simplicity. Exosomes and circulating tumor cells are regarded as potential liquid biopsy specimens. Exosomes only contain partial information about cancers (Li et al., 2017), and the isolation methods for circulating tumor cells are still challenging because of the extremely low number of such cells (Wang C. et al., 2015; Wang J. D. et al., 2015; Ruzycka et al., 2019). Relevant research is still in its infancy, but the analytical method of histopathologic biopsy can also be applied to liquid biopsies.

Reverse transcription (RT)-PCR is widely used in mRNA detection for its high sensitivity and specificity. However, RT-PCR cannot meet the requirements of rapid diagnosis because multiple temperature cycles are required, necessitating a lengthy detection time and a large real-time fluorescence detector (Maeda et al., 2009). Alternatively, as a type of nucleic acid amplification method performed under isothermal conditions, loop-mediated isothermal amplification (LAMP) could simplify the need for supporting equipment (Notomi et al., 2000). Thus, we developed a miniaturized microfluidic chip system to enable LAMP reactions, which has been used for the detection of viruses and pathogens (Lin et al., 2019; Lin et al., 2021). Herein, we demonstrated the combined rapid and automatic detection of multiple lung cancer biomarkers on a microfluidic chip.

Several studies demonstrate that examining combinations of multiple biomarkers can improve sensitivity and specificity (Harmsma et al., 2013). For example, Li et al. (2016) showed that the joint detection of markers such as CYFRA21-1, NSE, CEA, CYFRA21-1, CA125, and SCC further enhances diagnosis efficacy. Liu et al. (2017) suggested that the combination of CEA, CYFRA21-1, SCC, NSE, ProGRP, and CA125 can discriminate the histological types of lung cancer. However, every study has its self-defined cutoff levels for different reaction conditions and subjects, and the results are difficult to apply to other districts or countries. Compared to manual analysis, machine learning-based technology can avoid the interference caused by personal experiences and determine potential correlations with broader applicability. In the past few years, machine learning has been widely used in the field of biomedical research (Litjens et al., 2017), including discovering new biomarkers for lung cancer diagnosis (Xie et al., 2021).

Herein, we introduced a machine learning model with discriminative feature selection and feature transformation by margin maximization to perform multi-biomarker analysis to obtain more accurate, reliable, and understandable predictions in a minimally invasive manner than conventional single biomarker analysis. To the best of our knowledge, this is the first investigation involving mRNA biomarkers in lung cancer diagnosis. From a point-of-care perspective, this method has various advantages such as simplicity, ease of use, low cost, and real-time results. More importantly, because of its painless and minimally invasive nature, we believe that our assessment system for lung cancer will simplify physical examination processes and significantly improve patients’ medical experiences.

## 2 Materials and methods

### 2.1 Subjects

A total of 86 subjects were recruited at the Emergency General Hospital (Beijing, China) from March 2017 to January 2019. Included patients were stage II to stage IV. Patients with other unrelated diseases were excluded from the subject group to eliminate errors caused by irrelevant factors, with the exception of the pulmonary metastasis group. Patients with different malignancies, such as esophageal cancer and thyroid cancer, were included in the pulmonary metastasis group. The clinical features of the subjects are listed in Table 1. The research protocol was approved by the Ethics Committee of the Emergency General Hospital and Tsinghua University. All participants provided written informed consent before participating in this study. The tissue sample from each subject was obtained through bronchoscopy for mRNA expression analysis. All diagnoses were confirmed by traditional pathological examination by experienced clinicians at the hospital who were blinded to this study. The molecular analysis of all tissue samples in this study was performed at Tsinghua University (Beijing, China).

**TABLE 1:** Clinical features of subjects.

Variable	Number of subjects	Percentage (%)
Subjects with clinical features	86	100
Age (median 60, range 14–88)		
≤60	42	48.8
>60	44	51.2
Gender		
Male	58	67.4
Female	28	32.6
Histology		
Benign	20	23.3
Adenocarcinoma	15	17.4
Squamous carcinoma	28	32.6
SCLC	6	7.0
Pulmonary metastasis	17	19.7

### 2.2 mRNA expression analysis and the design and screening of loop-mediated isothermal amplification primers for the eleven lung cancer biomarkers

Carcinoembryonic antigen (CEA), cytokeratin fragment 19 (CYFRA21-1), squamous cell carcinoma antigen (SCC), neuron-specific enolase (NSE), and pro-gastrin-releasing peptide (ProGRP) are serological markers for lung cancer recommend by the American Committee on Clinical Biochemistry, the European Expert Group on Tumor Markers, and the Chinese diagnostic and therapeutic specifications for primary lung cancer (2015 Edition). Joint use can improve specificity and sensitivity in clinical application. Among them, NSE and ProGRP are ideal indicators for diagnosing SCLC (Li et al., 2022), CEA, CYFRA21-1, and SCC are helpful for the auxiliary diagnosis of NSCLC (Mishra et al., 2021), and CYFRA21-1 and SCC are considered to be specific for squamous cell carcinoma (Fatica et al., 2022). Another six biomarkers, namely, carcinoma antigen 125 (CA125) (Yang et al., 2018), epidermal growth factor receptor (EGFR) (Scharpenseel et al., 2019), isocitrate dehydrogenase 1 (IDH1) (Mishra et al., 2021), thyroid transcription factor-1 (TTF-1) (Liu et al., 2018), synaptophysin (SYN) (Wang et al., 2020), and neural cell adhesion molecule (CD56) (Svajdler et al., 2019) were selected according to their frequency of being mentioned in the relevant literature. Thus, 11 lung cancer biomarkers were chosen through extensive literature research in total. A maximum amount of 30 mg tissue sample was stored in RNAlater™ Stabilization Solution (Ambion, United States) immediately after harvest. After disruption and homogenization with a tissue lyser (DHS, China) and a stainless steel bead, the mRNA was extracted using an RNeasy Mini Kit (Qiagen, Germany) according to the manufacturer’s instructions.

**FIGURE 1:**
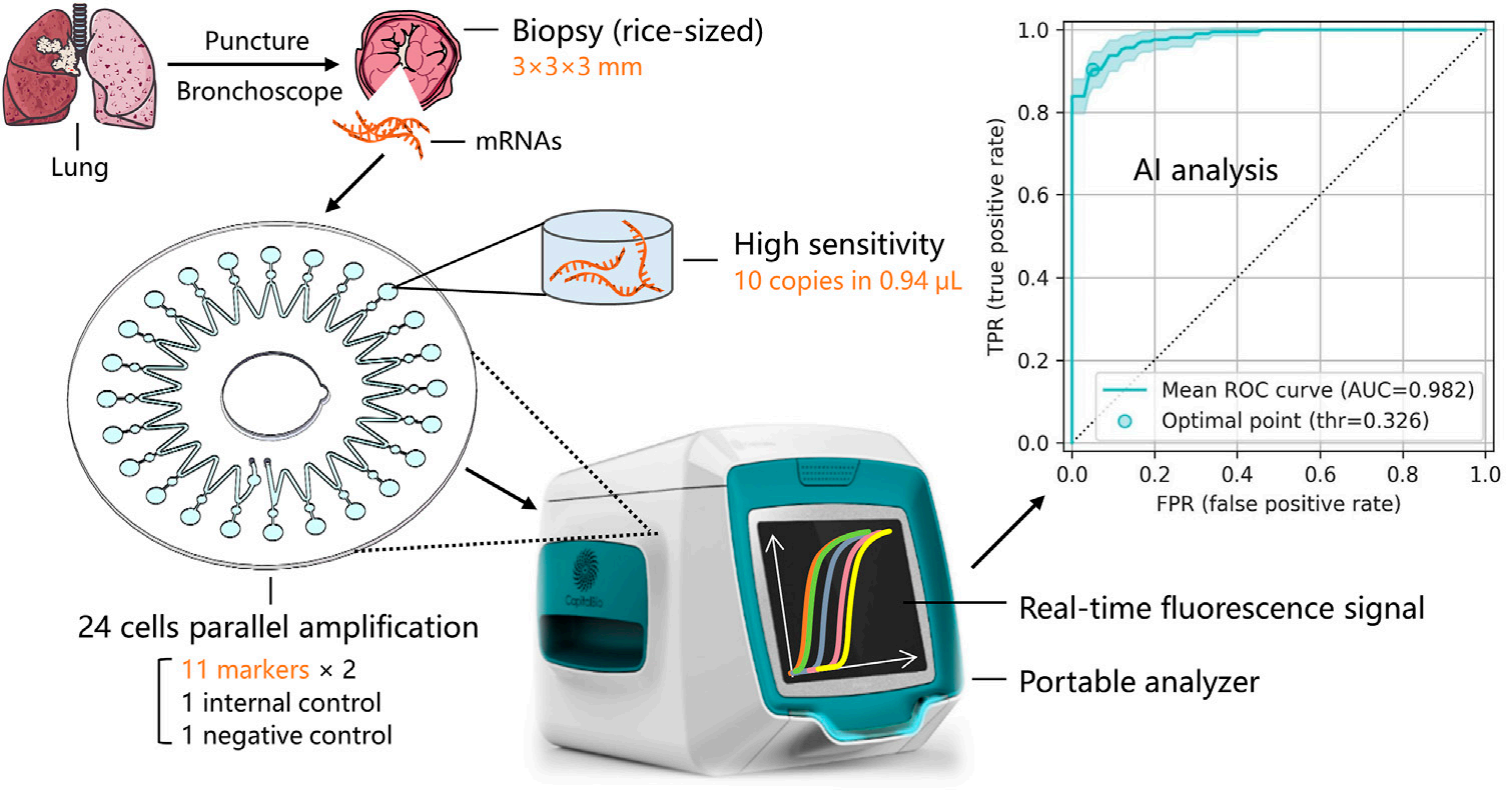
Sample acquisition and mRNA expression detection of multiple lung cancer typing biomarkers via a microfluidic chip and miniaturized fluorescence analyzer.

The mRNA levels of the 11 biomarkers in the tissue samples were measured by RT-LAMP, as shown in Figure 1. The RT-LAMP was performed with a WarmStart LAMP Kit (DNA&RNA) (New England BioLabs Inc., China) according to the manufacturer’s instructions. The reaction mixture (28 µL) contained 14 µL of WarmStart LAMP 2 × Master Mix, 0.56 µL of fluorescent dye (50 × ), and 13.44 µL of mRNA. The RT-LAMP reaction was performed on the microfluidic chip.

LAMP primers for the 11 lung cancer biomarkers were designed using the LAMP primer design software Primer Explorer version 4 (https://primerexplorer.jp/elamp4.0.0/index.html). A specific fragment of each biomarker was selected as the target by comparing related gene sequences in the NCBI (National Center for Biotechnology Information). First, two outer primers (F3 and B3) and two inner primers (FIP and BIP) were designed, and then two loop primers (LF and LB) were additionally designed to accelerate the reaction rate. The specificities of these primers were verified by Basic Local Alignment Search Tool (BLAST) analysis (Version 4, Bethesda, MD, United States), and the formation of primer dimers was assessed and excluded in primer design. After these steps, the LAMP primers for the 11 lung cancer biomarkers were synthesized by Sangon Biotech (Shanghai, China). Every set of primers was double-checked by practical experiments, including the limit of detection (LOD) analysis, linearity analysis, and specificity analysis. Only primer sets that passed all of the tests and performed best were chosen for each of the 11 biomarkers. Among them, the sequences of the LAMP primers for ProGRP are listed as a sample in Table 2. For ProGRP, four primer sets were first designed by the software, and three of them passed the BLAST test. All three primer sets were then synthesized, and the primers listed in Table 2 were finally chosen after practical experiments.

**TABLE 2:** ProGRP LAMP primer sequences.

Primer name	Sequence (5′-3′)
ProGRP-F3	GCT​GAC​CAA​GAT​GTA​CCC​G
ProGRP-B3	ACGAAGGCTGCTGATTGC
ProGRP-FIP	CTC​AGC​TGC​TGC​TTC​AGG​CTC-TGG​GGC​ACT​TAA​TGG​GGA
ProGRP-BIP	ACA​TCA​GGT​GGG​AAG​AAG​CTG​C-GGC​TGG​TGG​TTT​CTG​TTC​T
ProGRP-LF	GAA​ACA​GAA​GAA​GAC​TCC​CCT​G
ProGRP-LB	GCT​GGG​TCT​CAT​AGA​AGC​AAA​G

### 2.3 Miniaturized microfluidic chip system

**FIGURE 2:**
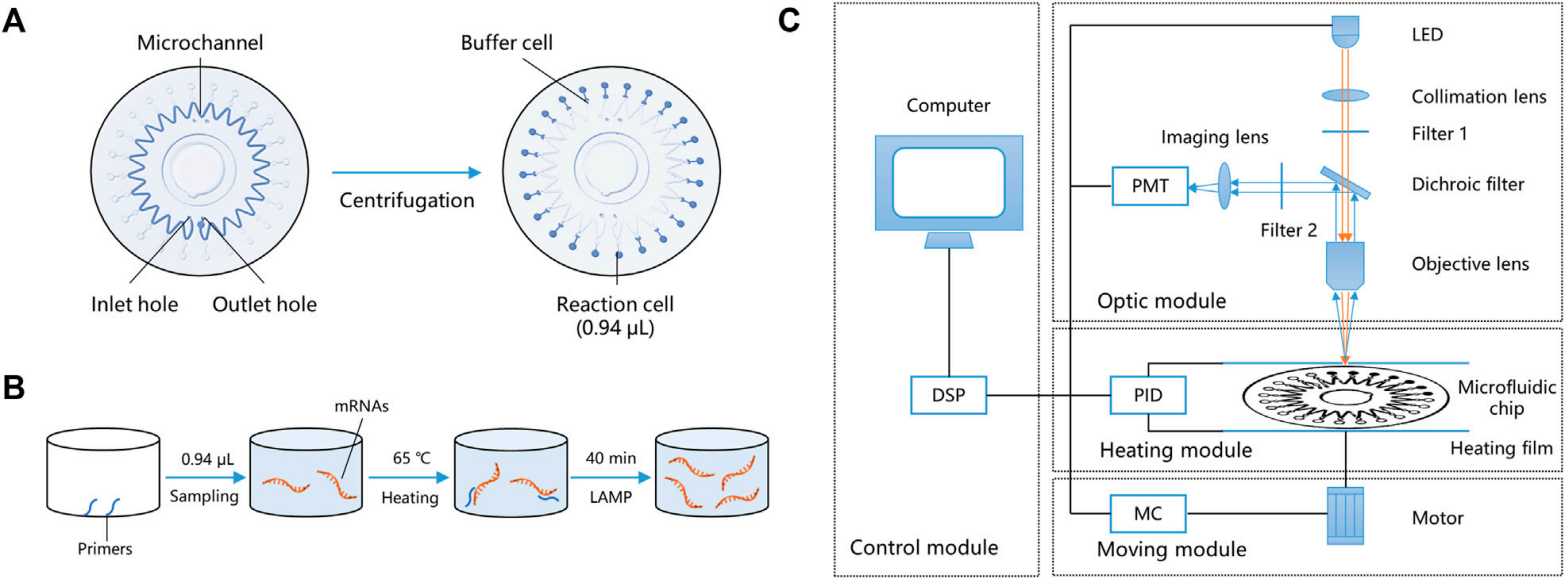
Working principle of the miniaturized microfluidic chip system. **(A)** Structure and liquid distribution of the microfluidic chip. **(B)** Reaction principles of LAMP in a bioreactor cell. **(C)** Schematic of the miniaturized real-time fluorescence detector. DSP: Digital signal processor; PMT: Photo-multiplier; LED: Light-emitting diode; PID: Proportional integral derivative; and MC: Moving controller.

The miniaturized microfluidic chip system contains two parts: a microfluidic chip and a miniaturized real-time fluorescence detector. The microfluidic chip for the parallel detection of multiple markers in a microliter reaction system after only one sampling was designed and developed. It could evenly distribute reaction reagents to 24 bioreactor cells using centrifugal force, and the sinusoidal shape of the microchannel enabled the prevention of cross-contamination. The microfluidic chip comprised two polycarbonate (PC) layers—a substrate layer and a top layer. The substrate layer contains microstructures such as channels and cells, and the top layer could be adhered to the substrate layer with double-sided tape. The radius of the microfluidic chip is 31 mm, and the total thickness is 1.2 mm. Figure 2A shows the structure of the microfluidic chip, including inlet and outlet holes, a microchannel, and 24 reaction cells and buffer cells. The reaction mixture was added to the chip through the inlet hole to fill the microchannel. Air was expelled through the outlet hole, and the chip was sealed by sticking a single-sided adhesive film to the inlet and outlet holes, forming a closed reaction system. After centrifugation, the reaction mixture was automatically distributed into the 24 reaction cells, and buffer cells were designed to hold the spare liquid and bubbles. In this way, 24 independent reactions could simultaneously be performed on the microfluidic chip. The adjacent buffer cells were isolated because of the sinusoidal shape of the microchannel, and after heating, a high-pressure air microchannel was formed, effectively avoiding cross-contamination caused by liquid diffusion. The volume of a bioreactor cell was only 0.94 μL, which is < 1/25th of the conventional 25-μL EP tube PCR method.

LAMP primers targeting the mRNAs of the 11 typing biomarkers and one internal reference (glyceraldehyde-3-phosphate dehydrogenase, GAPDH) were designed and pre-embedded with 0.1% agarose on the bio-reactor cells of the microfluidic chip. The chip’s 24 bioreactor cells contain two repetitions of the 11 typing biomarkers, one internal GAPDH reference, and one negative quality control. After sampling and heating to 50°C, the low melting point agarose melted, and the pre-embedded primers were released into the reaction mixture. The primers participated in the LAMP reaction at 65°C, while agarose had no impact on the amplification (Figure 2B). During isothermal amplification of the nucleic acid, the products of specific sequences were continuously generated and the fluorescent dye EvaGreen combined with the products, generating a green fluorescence signal. The fluorescence signals in the 24 reaction cells were detected and analyzed using the miniaturized real-time fluorescence detector, and real-time results were dynamically displayed.

As shown in Figure 2C, the miniaturized detector comprised four main parts: a control module, an optic module, a heating module, and a moving module. Users can input orders using a computer and instruct the other three modules using a digital signal processor (DSP). The control model is also capable of data acquisition and processing, including parameter setting, temperature control, and moving control. The moving module includes a three-dimensional motion platform (translation, rotation, and lifting), a rotating motor, and a multi-axis motion controller, which is controlled by an STM32 microprocessor. It could control the rotation of the microfluidic chip, align each bioreactor cell to the objective lens, and cooperate with the optical module to collect the real-time fluorescence signal. The heating module includes a proportional integral derivative (PID) temperature controller, two heating films, and temperature sensors. The two heating films made a double-sided 250 µm thin-layer air bath, heated the microfluidic chip to 65°C, and kept the temperature constant for 40 min for the LAMP reaction. The double-sided thin-layer air bath heating sped up the heat transfer efficiency and was suitable for scanning detection during the rotation of the microfluidic chip.

The optic model includes an incident light path and a fluorescent receiving light path. A schematic diagram of the optic model is shown in Figure 2C. In the incident light path, the exciting light source was a 1-W blue light-emitting diode (LED) (450–475 mm). The exciting light was concentrated by the collimation lens and filtered by filter 1 (463 ± 10 nm), directly passed through the dichroic filter, and focused on a reaction cell by the objective lens. In the fluorescent receiving light path, the fluorescent signal produced by the LED light was collected by the objective lens and reflected by the dichroic filter. Here, the function of the dichroic filter is to separate the two light paths and improve the use of light. Finally, the fluorescent signal was filtered by filter 2 (520 ± 20 nm) and focused on the photomultiplier (PMT) (Hamamatsu Photonics, Japan) by an imaging lens. Simultaneously, the real-time fluorescence signal was displayed on the computer (Lin et al., 2019). All of the raw data were normalized and represented the relative mRNA expression levels in each subject.

### 2.4 Statistical analyses

Statistical analyses were performed using Microsoft Excel. The correlation of mRNA expression with different clinical features was analyzed using Student’s *t*-test. All statistical analyses were two-sided, and a significance level of *P*< 0.05 was used. The cutoff values were decided by comparing the mRNA expression levels of the 11 biomarkers with different histology, and the mRNA expression levels of each biomarker were compared to the cutoff value of that biomarker. The status of each biomarker was considered high expression if the mRNA level was equal to or exceeded the cutoff value. Otherwise, it was considered a low expression.

To evaluate the performances of the biomarkers, sensitivity and specificity were calculated and compared. Sensitivity is defined as true positive/(true positive + false negative), and specificity is defined as true negative/(true negative + false positive). By summarizing sensitivity and specificity at different threshold levels, a receiver operating characteristic (ROC) curve (Fawcett, 2006) was plotted, and the AUC was calculated. The machine learning model was estimated by the AUC value.

### 2.5 Development of the machine learning model

**FIGURE 3:**
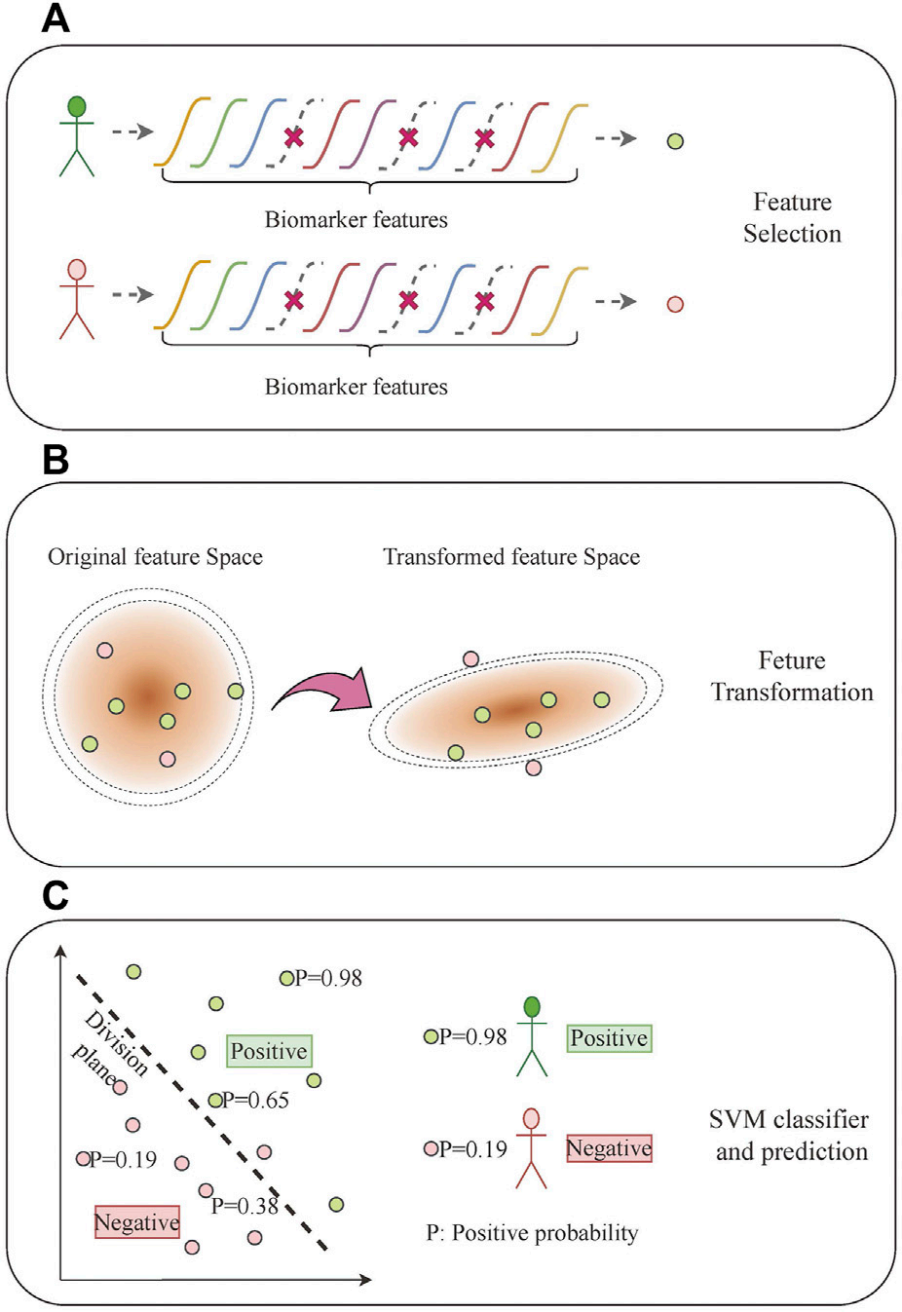
Multi-biomarker typing analysis based on machine learning. **(A)** Feature selection. **(B)** Feature transformation. **(C)** SVM classifier and prediction.

To analyze the relationship between the mRNA expression of multiple biomarkers and different subtypes of lung cancer, we proposed a rapid diagnosis model with discriminative feature selection and feature transformation by margin maximization. This machine learning-based model jointly takes all of the biomarker features as inputs and comprises three steps: recursive feature elimination (RFE)-based feature selection (Guyon et al., 2002), large margin nearest neighbor (LMNN)-based feature transformation (Weinberger and Saul, 2009), and support vector machine (SVM)-based classification (Cortes and Vapnik, 1995) (Figure 3). First, considering that not every biomarker feature counts in distinguishing each lung cancer subtype, the feature selection eliminates one feature at a time to recursively seek the significant biomarker features. Then, the feature transformation optimizes a linear transformation matrix to maximize the high-dimensional distance among different subtype samples and minimize the ones from the same subtype, which transforms the biomarker features into another feature space, making it easy to train the classifier. Finally, we used an SVM model as the classifier to build the final lung cancer subtype prediction.

Specifically, all of the 11 mRNA expression biomarkers comprise a 22-dimensional input feature ${\left\{X_{c}^{\left(i\right)}\right\}}_{c=1}^{22}$ for the *i*-th subject whose subtype class is $y^{\left(i\right)}$ , in which we incorporate the mean and standard deviation of the signal for each biomarker from the acquisition procedure. The feature selection module of the model takes *X* as the input and recursively eliminates one marker feature at a time, which results in ${\left\{S_{c}\right\}}_{c=1}^{s}$ , which varies from 2 to 22. The LMNN (Weinberger and Saul, 2009) feature transformation module optimizes a linear transformation matrix M :

$$M=\arg\underset{M}{min}\underset{i,j\in N_{i}}{\sum }∥M\left(S^{\left(i\right)}-S^{\left(j\right)}\right){∥}^{2}+\underset{i,j\in N_{i},y\left(i\right)\neq y\left(l\right)}{\sum }\max\left(0,1+∥M\left(S^{\left(i\right)}-S^{\left(j\right)}\right){∥}^{2}-∥M\left(S^{\left(i\right)}-S^{\left(l\right)}\right){∥}^{2}\right),$$

where $\text{j}\in {\text{N}}_{\text{i}}$ means $S^{\left(j\right)}$ is one of the k-nearest neighbors of $S^{\left(i\right)}$ , and they share the same class, that is, $y^{\left(i\right)}=y^{\left(j\right)}$ . M is initialized using principal component analysis (PCA) (Pearson, 1901).

The feature transformation module transforms the s-dimensional feature ${\left\{S_{c}\right\}}_{c=1}^{s}$ into an $n_{c}$ -dimensional feature ${\left\{F_{c}\right\}}_{c=1}^{n_{c}}$ . We set $n_{c}$ to five in our experiments. Then, we used an SVM classifier to perform lung cancer subtype classification using F. A support vector machine (Cortes and Vapnik, 1995) constructs a dividing hyperplane in the original or transformed space of the input feature, which can classify the input sample by optimizing the problem:

$$\left(\omega ,b,\varsigma \right)=\arg\underset{\omega ,b,\varsigma }{\min}\frac{1}{2}\omega^{T}\omega +∁\underset{i}{\sum }\varsigma^{\left(i\right)},$$

$$s.t.\;y^{\left(i\right)}\left(w^{T}\phi \left(F^{\left(i\right)}\right)+b\right)1-\varsigma^{\left(i\right)},$$

$$\varsigma^{\left(i\right)}\geq 0,$$

where *w* and *b* construct the dividing plane, and *φ* is a linear or Gaussian kernel. We searched the kernel type and other hyper-parameters in our model using the grid-search method.

To best use all of the subjects in our experiment and explore the general performance of our model, we used three-fold cross-validation to split the dataset into training and test sets, which were repeated 10 times with different random partitions. The input feature of each biomarker was normalized using the natural logarithm before being fed into the model. The specific feature selection and linear transformation were based on the statistical analysis of all of the subjects. The output of the final SVM classifier was set to probability form, which means that the classification threshold can flexibly be adjusted to meet different sensitivity and specificity requirements in the application process.

## 3 Results

### 3.1 Limit of detection, linearity, and specificity of the system

**FIGURE 4:**
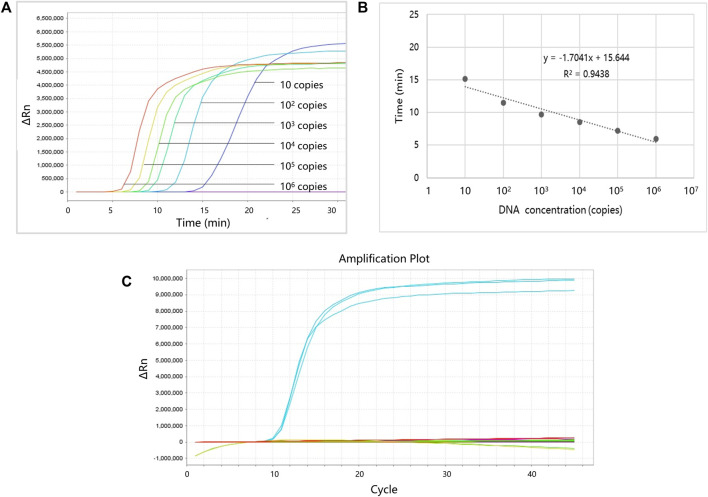
Limit of detection (LOD), linearity, and specificity analysis of ProGRP. **(A)** LOD analysis of ProGRP. **(B)** Linearity analysis of ProGRP. **(C)** Specificity analysis of ProGRP.

Serially diluted (10^6^, 10^5^, 10^4^, 10^3^, 10^2^, or 10 copies) plasmids were used to evaluate the performance of the LAMP primers. Each reaction was repeated three times, and the negative control group (using dH_2_O instead of plasmid) was also established. Figure 4 shows the LOD and linearity analysis of ProGRP. For the ProGRP primers, 10 copies of the plasmid could be detected using LAMP, indicating that the LOD was 10 copies. Similar to the cycle threshold (Ct) in a PCR, the time to positive in LAMP correlated with the concentrations of samples. As shown in Figure 4B, the standard curves of the ProGRP LAMP primers (three repeated experiments) display good linearity with a coefficient of determination (*R* ^2^) of 0.9438. For all of the 11 selected biomarkers, the LOD and linearity analyses of their LAMP primers were conducted in the same way. We found that the assay could detect as few as 10 copies of NSE, ProGRP, and CD56, and 10^2^ copies of the other eight biomarkers. All of the biomarkers displayed good linearity with an *R* ^2^ > 0.89, indicating the relative quantification reliability of the assay.

To verify whether the designed LAMP primers cross-react with each other, we also performed specificity verification analysis. The experimental conditions are the same as the aforementioned experiments, except that the templates corresponding to the 11 markers and water (negative control) were used to react with the LAMP primers of one marker each time, and all 11 primers were tested in turn. The template concentration was 10^5^ copies, and each experiment was repeated three times. Figure 4C shows the specificity analysis results of the ProGRP primers. They only reacted with the corresponding templates, and there was no reaction with other templates or self-amplification. The results of the other primer sets were the same, indicating that the primers we designed can amplify their targets specifically.

### 3.2 mRNA levels of the eleven biomarkers in different histology types

The mRNA levels of the 11 biomarkers were evaluated and compared among different histology types: benign, NSCLC (including LUAD and LUSC), SCLC, metastatic lung cancer, and primary lung cancer (including NSCLC and SCLC). Among these biomarkers, three were correlated with LUSC, one was correlated with metastasis, and five were correlated with SCLC. For CA125 and TTF-1, there were no significant expression differences observed between different histology types.

**FIGURE 5:**
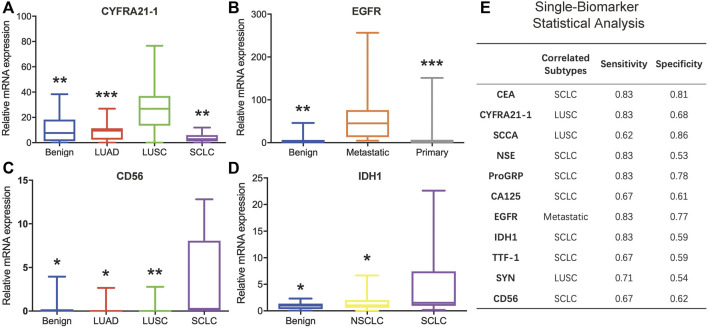
Analysis of the minimally invasive and rapid diagnostic method based on single biomarkers. **(A–D)** CYFRA21-1, EGFR, CD56, and IDH1 mRNA levels in different histology types. **(E)** Single-biomarker statistical classification analysis of the 11 biomarkers.

The mRNA expression levels of CYFRA21-1, SCCA, and SYN were correlated with LUSC. The CYFRA21-1 expression in LUSC was higher than in benign tissue (*p* = 0.003), LUAD (*p* = 0.001), and SCLC (*p* = 0.003) (Figure 5A). The SCCA expression in LUSC was higher than in LUAD (*p* = 0.031). On the contrary, the SYN expression in LUSC was lower than in benign tissue (*p* = 0.035) and SCLC (*p* = 0.028). The expression levels of EGFR were correlated with metastasis. mRNA expression increased in groups with metastatic lung cancer compared to the benign (*p* = 0.001) and primary lung cancer (*p* = 0.000) groups (Figure 5B).

The mRNA expression levels of CEA, ProGRP, CD56, IDH1, and NSE were correlated with SCLC. The CEA expression in SCLC was higher than in LUSC (*p* = 0.038). The ProGRP expression in SCLC was higher than in benign tissue (*p* = 0.030) and LUSC (*p* = 0.017). The CD56 expression in SCLC was higher than in the benign (*p* = 0.032), LUAD (*p* = 0.035), and LUSC (*p* = 0.004) groups (Figure 5C). The IDH1 expression in SCLC was higher than in benign tissue (*p* = 0.044) and NSCLC (*p* = 0.021) (Figure 5D). The NSE expression in SCLC was higher than in the benign (*p* = 0.005) and LUAD (*p* = 0.018) groups.

### 3.3 Single biomarker analysis

After determining the cancer subtype each biomarker could distinguish, all 86 clinical samples were classified according to each of the cutoff values of the 11 biomarkers. Briefly, for each biomarker, a high-mRNA expression sample was regarded as the specific subtype, and a low-mRNA expression sample was regarded as one of the other subtypes. Figure 5E shows the detailed classification performance of the 11 biomarkers, and the evaluation index includes sensitivity and specificity. CYFRA21-1 and SCCA could be combined to determine LUSC. EGFR could effectively distinguish metastatic lung cancer with high sensitivity and specificity. For SCLC, CEA and ProGRP displayed better performance than the other five markers. However, statistical analyses based on a single biomarker could not distinctly classify different subtypes.

CEA is recommended by the NACB (National Academy of Clinical Biochemistry) guidelines for NSCLC diagnosis when combined with CYFRA21-1, and IDH1 is used as a blood biomarker for the diagnosis of NSCLC^65^. However, in our study, the mRNA expression levels of CEA and IDH1 in SCLC were higher than in NSCLC. There may be several reasons for these results. First, the number of SCLC samples used in our study was small (only six). Second, SCLC is acknowledged to be more malignant than NSCLC. Finally and most importantly, other studies measured protein rather than mRNA levels, and mRNA expression is not completely synchronized with protein expression. Further studies should be conducted to confirm our results.

### 3.4 Multi-biomarker analysis based on machine learning

Jointly considering multiple biomarkers, we evaluated the performance of the introduced machine learning classification model for identifying different subtypes of lung cancer. The model was applied to five binary class settings: benign and malignant, adenocarcinoma and non-adenocarcinoma, squamous carcinoma and non-squamous carcinoma, SCLC and non-SCLC, and pulmonary metastasis and non-pulmonary metastasis. By summarizing the sensitivity and specificity at different threshold levels, an ROC curve (Fawcett, 2006) was plotted, and the AUC was calculated.

**FIGURE 6:**
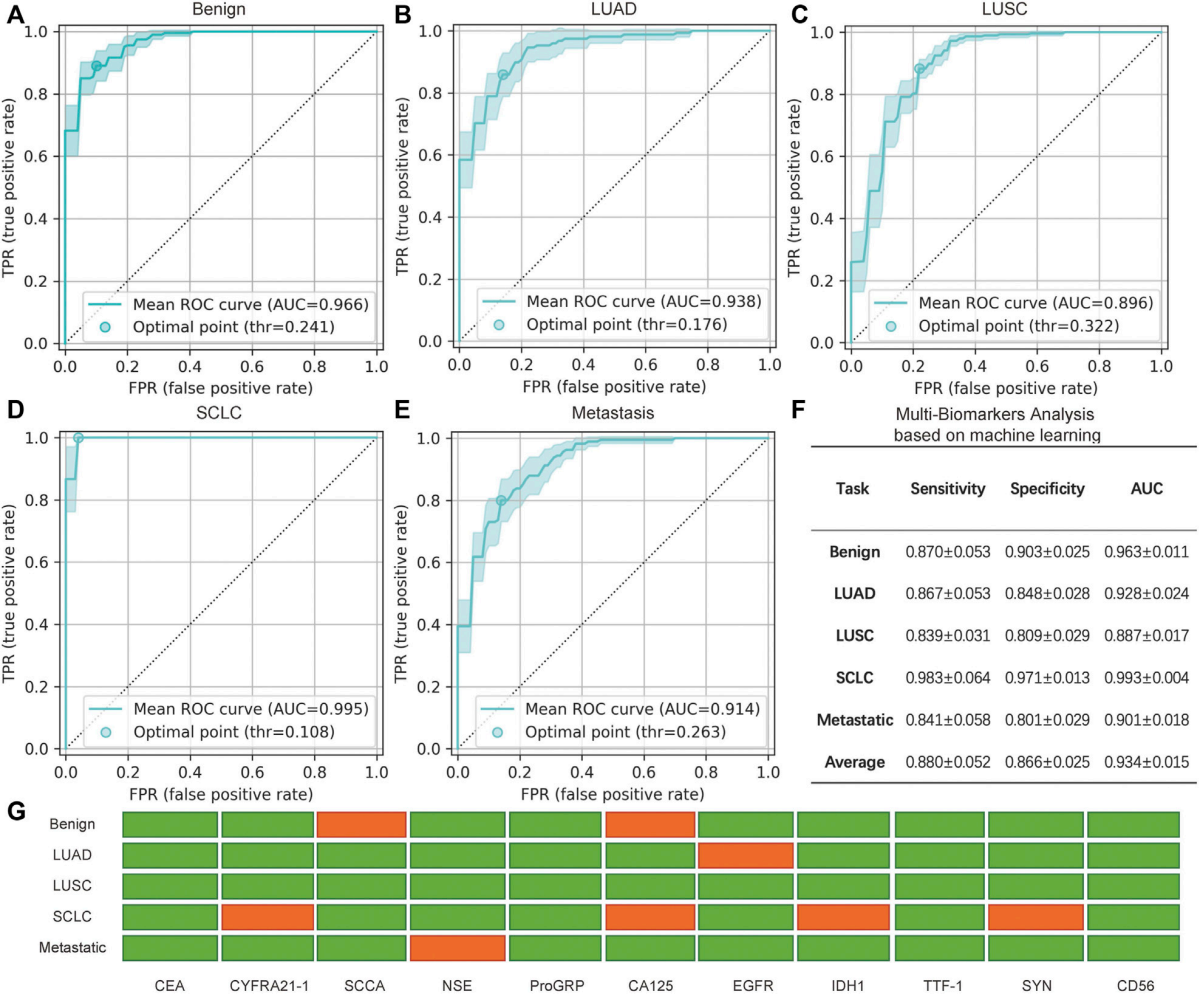
Analysis of the minimally invasive and rapid diagnostic method based on multiple biomarkers. **(A–E)** Mean receiver operating characteristic (ROC) curve with the 95% confidence interval (CI) and the optimal point for five binary multi-biomarker classification tasks based on machine learning. **(F)** Specific mean sensitivity, specificity, and AUC score for the five tasks, in which ± indicates the 95% CI. Note that the values in F are averages of all values with different optimal points of every cross-validation experiment and differ from the mean ROC curves in **(A–E)** that are averaged based on all curves. **(G)** Selected (green) and rejected (red) biomarkers generated by the feature selection module via recursive elimination. The selected biomarker features were fed into the feature transformation and the classifier modules to build the final prediction.

The performance of the five binary classification models is shown in Figures 6A–E. The results were averaged based on all of the test sets of the 10 repeats of the three-fold cross-validation procedure, which yielded 30 isolated experiments for each classification task. The ROC curves and the 95% confidence intervals (95% CI) are shown in Figures 6A–E, in which the optimal point is the threshold point that has the shortest distance to the upper left point in the ROC curve figure. In addition, the sensitivity, specificity, and AUC scores at the optimal point are shown in Figure 6F, in which the mean and 95% CI are given. The final result of the feature selection module is also shown in Figure 6G.

In general, benign and SCLC subjects were relatively easy to identify. A total of nine biomarkers were chosen to judge benign and malignant lung cancers, with an AUC score of 0.963; seven biomarkers could be used to distinguish SCLC from NSCLC, and the sensitivity was 0.983. Thus, this assay could accurately classify the main classes of lung cancer. Pulmonary metastasis could also be recognized by 10 biomarkers, including EGFR. Compared to single biomarker analysis with EGFR, combined analysis of the biomarkers increased the sensitivity and specificity. As for the classification of NSCLC, the AUC scores of adenocarcinoma and squamous carcinoma were slightly lower. However, all of the AUC scores are approximately 0.88 or greater, and the average score is 0.934. Therefore, this promising average performance suggests the validity of using mRNA biomarkers for the minimally invasive and rapid diagnosis of lung cancer. Although some biomarkers were not significant for specific binary classifications, the combination of multiple biomarkers was an improvement over the use of single biomarkers.

## 4 Discussion

By far, clinical lung cancer diagnosis techniques such as chest X-rays, CT scans, and pathological sectioning methods are the most widely used for lung cancer, yet none of them are sensitive and specific enough for the identification of new biomarkers. Although immunoassays are sensitive and selective for the early diagnosis of lung cancer, disadvantages remain because they are time-consuming, expensive, multistep, and often require large and expensive equipment. Despite the fact that molecular biology methods such as RT-PCR can be powerful to detect tumor cells, it should be noted that these methods require complicated manual operations, multiple instruments, and experienced operators to perform the analyses. In addition, methods based on EP tubes or multi-well plates consume >25 μL of samples and reagents, and thus, large amounts of tissue must be sampled. Furthermore, multiple samplings are also needed for typing, which is harmful to the operator and difficult to achieve, for many diseased tissues do not meet the requirements of multiple sampling.

In recent years, various types of factors, such as cell-free DNA, circulating tumor cells, and exosomes in bodily fluids, have been analyzed for the detection of lung cancer. Cell-free DNA and circulating tumor cells are present in very small amounts, resulting in the need for special collection devices, and expression levels are hard to obtain in most cases (Gao et al., 2017; Qian et al., 2018). Lung cancer-specific exosomal markers are still under study (Niu et al., 2019). Park et al. (2017) and Shin et al. (2020) classify exosomes by surface-enhanced Raman scattering and could distinguish cancer cell-derived and normal cell-derived exosomes with high sensitivity and specificity. However, non-specific detection of exosomes may lead to inaccurate diagnosis, and the major signals are only derived from surface molecules, which do not provide complete information.

Our assay uses histopathologic biopsy and detects mRNA biomarkers corresponding to protein tumor markers. Such multi-biomarker analysis based on machine learning is a general method and can be applied to liquid biopsies as well. For data acquisition, nucleic acid analysis based on the bronchoscope sampling method has obvious advantages. As a minimally invasive technique, it requires only rice-sized tissue samples. One sampling can detect 11 biomarkers at the same time and enable accurate typing and identification. The operation is also simple and rapid (within 45 min), with low cost for small typing reaction cells (only 0.94 μL), less than 1/25 of the conventional 25 μLEP tube test method.

AI is a powerful tool in biomedical research that is used to analyze deep features and connections among lesions and pathological disorders. In this study, we first used machine learning techniques to build a correlation graph from minimally invasive mRNA biomarkers to lung cancer subtypes. The model, jointly considering all mRNA biomarkers, was a great improvement over single biomarker analysis.

However, our study still has some challenges. First, the data scale in our study includes only 86 subjects, which may limit the performance of our models. Expanding the number of subjects would improve the robustness and reliability of our method. Second, the expression levels of mRNA biomarkers in the biopsy samples were first applied to the specific classification of lung cancer. Diagnosis based on protein is the traditional method and also the gold standard. Based on this view, it is more accurate. However, the detection of protein usually requires a large sample volume, complicated manual processing steps, and a lengthy time to generate final results. Furthermore, precise typing is still challenging in the clinic. Thus, we attempted to use mRNA as a new target and achieved quite good classification results, but further research should be performed to verify the assay. With larger datasets and improvements in deep learning technology in the future, we believe this study can be evaluated more thoroughly.

It is hoped that the minimally invasive and rapid AI diagnosis of lung cancer can be used as a guide to enter grassroots communities and even families to enable timely screening, long-term tracking detection, and health monitoring of lung cancer or other diseases. Once abnormalities are found, physicians can use minimally invasive methods to quickly perform multi-index typing and precise molecular diagnosis for personalized treatment. Then, the non-invasive method could be used to longitudinally track patients to evaluate the treatment effect.

## 5 Conclusion

Herein, we developed a rapid, minimally invasive, and convenient nucleic acid biomarker recognition method, which enabled the combined specific detection of 11 lung cancer typing markers in a microliter reaction system after only one sampling. This method uses a miniaturized microfluidic-based nucleic acid analyzer combined with deep learning and machine learning.

In this method, the primers for the combined detection of 11 lung cancer typing markers were designed and screened, the microfluidic chip for the parallel detection of the markers was designed and developed, and a miniaturized microfluidic-based analyzer was also constructed. We found that differences in the mRNA expression of multiple lung cancer typing biomarkers can be used to classify histology types from rice-sized tissue samples collected by bronchoscopy. Lung cancer subtypes could be identified within 45 min. The volume of each reaction cell was only 0.94 μL, and the sensitivity was as low as 10 copies with good linearity (*R* ^2^ = 0.9938). This significantly reduced the testing cost and significantly simplified the process of detecting multiple subtypes in parallel. To leverage multiple biomarkers together for the typing of lung cancer, a machine learning-based approach is proposed with discriminative feature selection and feature transformation by margin maximization. A total of 86 clinical samples were tested using the miniaturized nucleic acid analyzer and classified by the multi-biomarker analysis based on the machine learning model. The importance of each biomarker in identifying cancer subtypes was analyzed via a machine learning approach and achieved an average AUC of 0.934 on five binary classification tasks. This assay could distinguish benign from malignant lung cancer and also classify LUAD and LUSC in the NSCLC group. Moreover, the metastasis of lung cancer could be distinguished with an AUC score of 0.9. These promising results highlight the prospect of minimally invasive, rapid, and precise typing diagnosis of lung cancer using mRNA biomarkers in clinics.

## Data Availability

The original contributions presented in the study are included in the article, further inquiries can be directed to the corresponding authors.
